# Oncogenic osteomalacia: Problems in diagnosis and long-term management

**DOI:** 10.4103/0019-5413.69320

**Published:** 2010

**Authors:** Ish K Dhammi, Anil K Jain, Ajay Pal Singh, Puneet Mishra, Saurabh Jain

**Affiliations:** Department of Orthopedics, UCMS and GTB Hospital, Delhi, India

**Keywords:** Oncogenic osteomalacia, mesenchymal tumor, hypophosphatemic osteomalacia

## Abstract

Oncogenic osteomalacia is a rare association between mesenchymal tumors and hypophosphatemic rickets. It is more of a biochemical entity than a clinical one. The pathophysiology of the tumor is not clear. However, it has been seen that the clinical and biochemical parameters become normal if the lesion responsible for producing the osteomalacia is excised. For a clinical diagnosis a high index of suspicion is necessary. We present three such cases where in one the oncogenic osteomalacia reversed while in rest it did not. We present this case report to sensitize about the entity.

## INTRODUCTION

Oncogenic osteomalacia is a rare endocrinological paraneoplastic syndrome associated with large or very small mesenchymal tumors that apparently produce osteomalacia and biochemical abnormalities, including hypophosphatemia, normocalcemia, and increased levels of alkaline phosphatase.^1^–^8^ The pathophysiology of the tumor is not yet clear; however, a humoral factor produced by the tumor is suspected to be responsible for the osteomalacia.^2^,^3^ The removal of the ongoing oncogenic process leads to regression of the osteomalacia. The management can be problematic because the detection of a single and small lesion is very difficult. Moreover, when the patient has multiple lesions, it is difficult to know which lesions are producing/secreting osteomalacia-producing factors. Cases have been reported where the diagnosis has been delayed by up to 19 years,^9^ which suggests that the condition is poorly recognized. We present our experience with three such cases to draw attention to this condition and describe the problems associated with its diagnosis and management.

## CASE REPORTS

### Case 1

**Figure 1:**
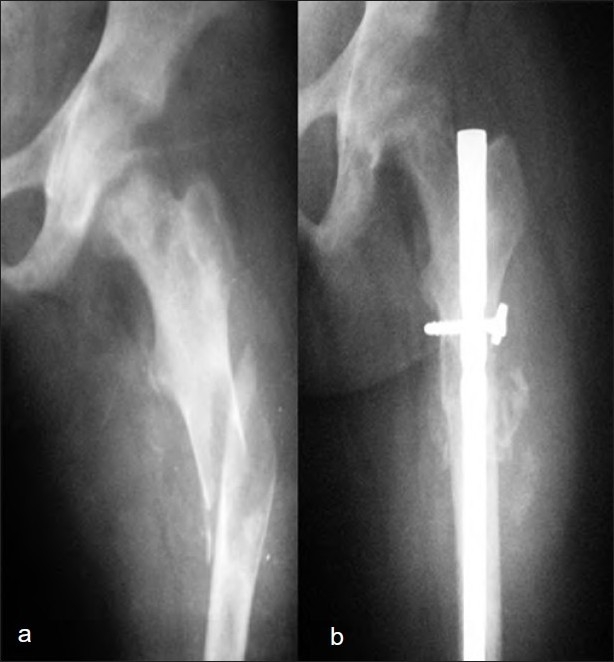
(a) Anteroposterior radiograph of femur showing lytic area with pathological fracture shaft femur. (b) At 1 year follow up radiograph showing union and nail *in situ*

A 16-year-old female was admitted with complaints of pain in the left thigh and inability to bear weight on the left leg since 1 day, following a trivial fall. Her left thigh was swollen, tender and demonstrable abnormal mobility, and crepitus was felt on attempted range of motion of hip examination. Radiographs of the femur showed a lytic area, with pathological fracture of the mid-shaft of the femur [Figure 1a]. The patient reported having had generalized bone pains for last 4 years. Her biochemical parameters showed the following: serum calcium 8 mg/dL (normal range: 8-11 mg/dL), serum phosphorus 1.7 mg/dL (normal range: 2.5-5.5 mg/dL), parathyroid hormone (PTH) 56 pg/mL (normal range: 15-65 pg/mL), alkaline phosphatase 436 IU (normal range: 150-250 IU), 24-hour urinary phosphorus 3466 mg% (normal range: 340-1300 mg%), and urinary calcium 230 mg% (normal range: 10-250 mg%). Thus, she had hypophosphatemia, normocalcemia, and raised alkaline phosphatase. Her skeletal survey did not reveal any abnormality. The laboratory investigations were suggestive of oncogenic osteomalacia. She was operated and curettage of the lesion was done. Interlock nailing of the femur and autologous corticocancellous bone grafting was done. Histopathology showed the lesion to be fibrous dysplasia. Postoperatively, she was given oral phosphate 500 mg thrice daily, oral elemental calcium 1 g twice daily and alpha-hydroxylated vitamin D 0.25 *μ*g twice daily. At follow-up after 2 years, the radiographs show sound union [Figure 1b]. Her metabolic profile returned to normal after 1 year of treatment and no derangement has been found till the last follow-up. All symptoms (bony pains) have disappeared and there has been complete healing of the fracture site and cavity; the biochemical parameters have also returned to normal at the last follow-up. Hence, calcium and phosphorus supplementation was stopped at 1 year.

### Case 2

**Figure 2:**
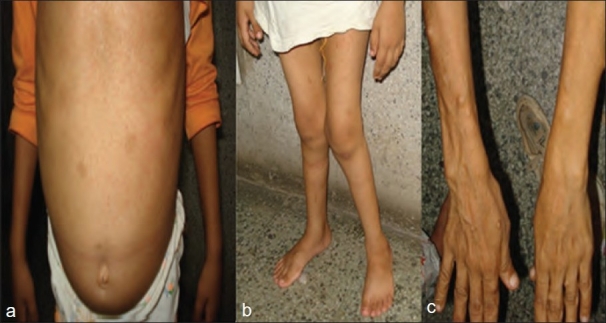
Clinical photograph of patient showing (a) Café au lait spots on abdomen. (b) Bilateral Genu valgum. (c) Clinical photograph of patient’s mother showing nodules on forearm

**Figure 3:**
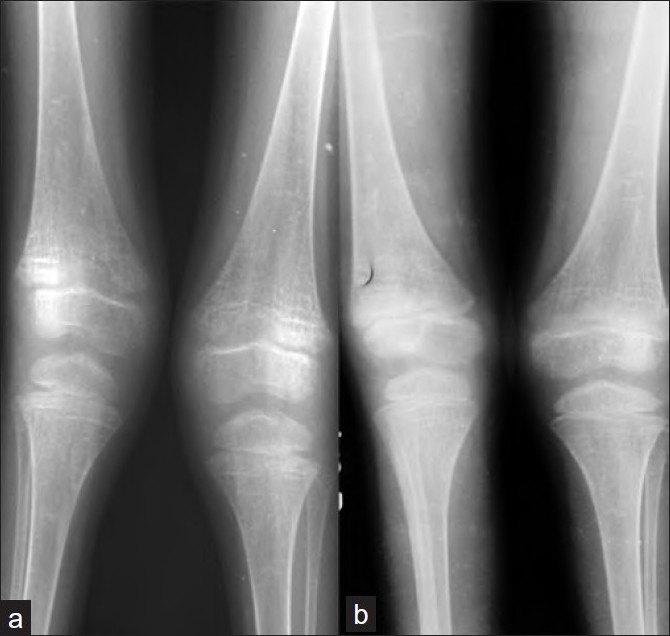
(a) Anteroposterior radiographs of both knee joints showing decreased bone density and trabeculations. (b) follow up radiograph at one year shows bone density has improved and trabeculations are less prominent

An 8-year-old female visited the outpatient department (OPD) with complaints of gradual-onset, generalized bone pains and deformity of both lower limbs (genu valgum) since 3 years. There was no preceding history of fever, trauma, or weight loss. On examination, she had bilateral genu valgum, widening of the wrist, frontal bossing, poor dentition, pectus excavatum, double malleoli sign, bony tenderness, significant *café au lait* spots on the back and abdomen [Figure 2a], blue sclera, latent nystagmus, and Lisch nodules. Her intermalleolar distance in the standing posture was 12 cm [Figure 2b]. X-ray of both wrists showed cupping, fraying, and widening of the growth plates. Her knee radiographs showed osteoporosis, with flaring and cupping of bilateral femoral metaphyses [Figure 3a]. Her mother is a known case of neurofibromatosis type 1 (NF 1) [Figure 2c]. Ultrasound for renal cysts was negative. Her biochemical parameters revealed serum calcium 8.2 mg/dL, serum phosphorus 1.8 mg/dL, alkaline phosphatase 440 IU, PTH 55 pg/ml, 24-hour urinary phosphorus 3678 mg%, and urinary calcium 200 mg%. Thus, she had hypophosphatemia, normal serum calcium, and raised alkaline phosphatase. A diagnosis of neurofibromatosis (NF) type 1 with oncogenic rickets was made. She was treated with 6 lakh units of vitamin D intramuscularly, followed by supplementation with calcium 1 g and vitamin D 0.25 mg daily, along with 500 mg oral phosphate thrice daily.

At 18 months’ follow-up her bone pains are controlled and the intermalleolar distance has been reduced to 11 cm. X-ray shows evidence of healing rickets [Figure 3b]. Her biochemical parameters are slightly improved though not yet normal [Table 1]. She is still on calcium and phosphate supplementation at follow-up.

**Table 1:** Laboratory values at 1 month, 2 month and 1 year in our cases

Values	Patient 1			Patient 2			Patient 3								
	1 month	2 months	1 year	1 month	2 months	1 year	1 month	2 months	1 year
Serum calcium (mg%)	8	8.8	10	8.2	8.4	8.6	8.6	8.7	8.8
Serum phosphorus (mg%)	1.7	2.3	3.0	1.8	1.9	1.8	1.9	2 2
Alkaline phosphatase (IU)	436	300	226	440	436	430	445	440	440
PTH (pg/mL)	56	54	54	55	52	54	51	53	54
24-hour urinary phosphorus (mg%)	3466	2246	1256	3678	3600	3646	4845	4800	4812
Urinary calcium (mg%)	230	216	210	200	206	186	181	180	170

### Case 3

A 38-year-old female presented to the emergency department with complaints of pain in the left hip and inability to bear weight for 2 days, following taking a sharp turn while walking. Clinical examination revealed swelling, tenderness, bruises, abnormal mobility and crepitus around a subtrochanteric area. Radiographs of the left hip and femur showed a pathological subtrochanteric fracture, with a lytic lesion in the proximal femur [Figure 4A]. The patient gave a past history of generalized weakness, off and on bone pains, muscular pains, and chronic fatigue for the last 12 years. Her skeletal survey revealed looser zones in the pubic rami and osteolytic lesions in the ribs and scapulae [Figure 4B]. Her biochemical parameters revealed serum calcium 8.6 mg/dL, serum phosphorus 1.9 mg/dL, alkaline phosphate 445 IU, parathyroid hormone (PTH) 51 pg/mL, 24-hour urinary phosphorus 4845 mg%, and urinary calcium 181 mg%. Thus, she had hypophosphatemia, increased alkaline phosphatase, and hyperphosphaturia, with normal calcium and parathormone levels. A clinical diagnosis of oncogenic osteomalacia and polyostotic fibrous dysplasia with pathological fracture of the proximal femur was entertained. The patient was taken up for curettage of the lytic lesion, bone grafting, and internal fixation. Peroperatively, the cortex of the femur was seen to be papery thin and surrounding a large (50 mm ^3^ volume) cavity filled with high straw/brown-colored fluid. The cavity had few membranes which were curetted out. The cavity was then packed with autologous fibular grafts and corticocancellous grafts. The fracture was secured with a dynamic compression screw and plate. She was kept on skeletal traction for 8 weeks and then mobilized on ischial weight-relieving calipers and axillary crutches. Postoperatively she was given oral phosphate 500 mg thrice daily, oral elemental calcium 1 g twice daily, and alpha-hydroxylated vitamin D 0.25 *μ*g twice daily. Histopathological examination showed fibrous dysplasia. At follow-up at 1.5 years the fracture had united [Figure 4B] and laboratory investigations revealed serum calcium 8.8 mg/dL, serum phosphate 2 mg/dL, and alkaline phosphatase 440 IU. Phosphates level had improved but was not yet normal [Table 1]. The patient was mobile without support. The patient was still on calcium and phosphate supplementation at the last follow-up.

**Figure 4A:**
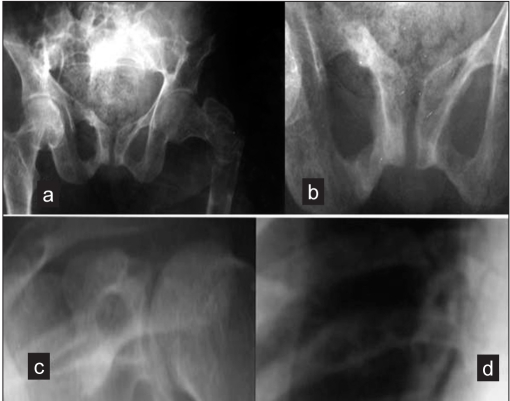
(a) Anteroposterior radiograph of pelvis with both hip joints showing a pathological subtrochanteric fracture with lytic lesion in proximal femur. (b) Loosers zones in pubic rami. (c) Osteolytic lesion in scapula. (d) Osteolytic lesion in ribs

**Figure 4B:**
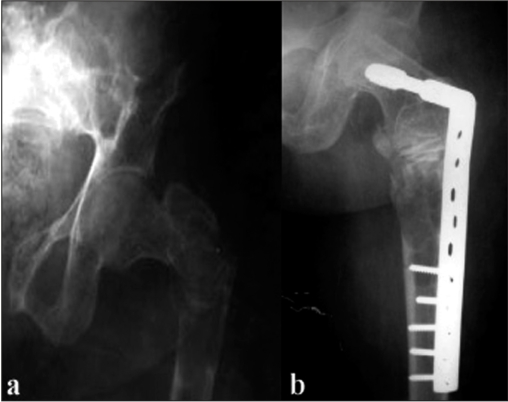
(a) Pre-operative anteroposterior radiograph showing pathological subtrochanteric fracture. (b) Postoperative follow-up anteroposterior radiograph showing DCS fixation with fibular and cortico cancellous grafts *in situ*

## DISCUSSION

Oncogenic osteomalacia is a term given to a rare endocrinological paraneoplastic syndrome characterized by defective bone mineralization as a result of renal phosphate loss. McCance reported the first case of oncogenic osteomalacia in 1947 in the distal femur of a 17-year-old girl.^5^ The relationship between these mesenchymal tumors and osteomalacia was first described by Prader *et al*. 40 years ago.^1^ Since then more than 100 cases have been reported.^6^ It is an unusual condition but probably still is the most common cause for acquired hypophosphatemic osteomalacia in adult males.^3^ The oncogenic cause of osteomalacia may remain unrecognized as the tumors are frequently small and asymptomatic.^1^ Hemangiopericytoma, giant cell tumor, nonossifying fibroma, and hemangioma are the most common histological entities.^3^

The age distribution of patients ranges from 7 to 77 years, with the male:female ratio being 1.2:1. The onset is gradual and the patient presents with joint deformities, bone pain, waddling gait, muscle weakness, fatigue, anorexia and, rarely, long bone fractures. The clinical course is protracted.^1^ Clinical signs appear from several months to years before the discovery of the tumor in most of the patients, while in some cases a neoplastic mass is noted long before the onset of skeletal disease; thus, a high index of suspicion is required to diagnose this entity.^4^ In two of our cases the patients presented with pathological fractures of the lower extremity and the onset of skeletal symptoms was long before the pathological fractures occurred, whereas in the other case (case 2) it presented with deformity in the lower extremity and oncogenic osteomalacia was detected at that time.

The metabolic picture of this syndrome shows hypophosphatemia, hyperphosphaturia, low or normal serum calcium, raised alkaline phosphatase, normal PTH, low concentrations of 1, 25-dihydroxycholecalciferol, decreased tubular resorption of phosphates,^1^,^3^ low urinary calcium, and increased urinary phosphate. Radiographic findings may reveal multiple pseudofractures, indistinctiveness or loss of trabecular structure, and nonspecific decrease in bone radiodensity. The search for the possible tumor should start with the clinical examination. A skeletal survey may help in localizing the lesion. Various modalities have been used to detect the lesions responsible for oncogenic osteomalacia, including CT, MR, whole-body ^99^mTc-sestamibi scan^111^, octreotide scintigraphy,^201^ TI scintigraphy, ^99m^Tc-MIBI SPECT, and venous sampling for FGF23 with MR imaging.^10^ Hypophosphatemic osteomalacia may be evident when the tumor has already become obvious as happened in all our three cases. If the patient is unaware of the tumor and a thorough clinical examination fails to detect such a lesion, then scintigraphy may be helpful. These tumors have prominent vasculature in 50% of cases and hence scintigraphy with a prominent blood-pool phase and angiography may be helpful. No data is available proving the usefulness of CT/MRI.

In the two cases of fibrous dysplasia, one was polyostotic while the other was solitary. We did not have bone scan to document the polyostotic nature, however skeletal survey was done in every case and revealed fibrous dysplasia in a rib and the scapula in case 3. PET/SPECT can be helpful in cases of polyostotic fibrous dysplasia when the lesion inducing oncogenic osteomalacia is not known.

In oncogenic osteomalacia the biochemical and clinical abnormalities are caused by a circulating factor produced by the neoplasm. Tumor extracts injected in dogs, mice, and rats caused phosphaturia in experimental studies.^11^,^12^ The circulating factor is named phosphatonin. Patients of autosomal dominant hypophosphatemic rickets (ADHR) have clinical and laboratory similarities to oncogenic osteomalacia. The ADHR gene is a polypeptide that causes a phosphate-wasting disorder and is identified as fibroblast growth factor (FGF-23).^13^–^15^ FGF-23 has all of the predicted biological properties of phosphatonins.^13^,^14^ High levels of FGF-23 have been demonstrated in tumors from patients with oncogenic osteomalacia and X-linked hypophosphatemia. Its levels increases in oncogenic osteomalacia and diminishes after removal of the tumor. In cases of fibrous dysplasia, renal phosphate wasting has been linked to the overproduction of FGF-23 by mutated osteoblasts.^7^ FGF-23 is readily detectable in the plasma or serum of healthy individuals and is markedly raised in oncogenic osteomalacia. FGF-23 levels assessed by ELISA can aid early diagnosis of oncogenic osteomalacia.^3^ Cultures from oncogenic osteomalacia–associated tumors have identified one more protein FGF-7 – a potent and direct inhibitor of phosphate uptake *in vitro*, while only small amounts of FGF-23 were present. FGF-7 derived from oncogenic osteomalacia–producing tumor cultures was confirmed to be potent inhibitor of phosphate transport *in vitro*.^8^ Two other proteins responsible for producing humoral factors - MEPE (matrix extracellular phosphoglycoprotein) and FRP4 (secreted frizzled-related protein) have also been described. These proteins are expressed by tumors in oncogenic osteomalacia.^4^

The treatment of choice in oncogenic osteomalacia is tumor removal.^1^–^6^ It leads to a dramatic improvement in the clinical course of the disease as well as in the serum biochemical markers of bone turnover. Serum phosphate levels and TMP/GFR of phosphate return to normal within hours to days after tumor removal.^3^ Therapy is aided by short-term replacement of phosphate orally and addition of vitamin D and calcium. One of our cases had complete recovery and we were able to stop treatment.

However, the problems in management continue if one is not able to detect the oncogenic osteomalacia–producing tumor. Hence, one has to continue with calcium and phosphorus supplements, probably throughout life. With this treatment the clinical symptoms like bone pains, fatigue, and lethargy remains well controlled, while the biochemical parameters never return to normal. This happened in two of our cases in which polyostotic fibrous dysplasia and neurofibromatosis were the diagnoses. In cases of diffuse tumor leading to this condition, high doses of calcitriol and oral phosphate are indicated as in the treatment of X-linked hypophosphatemia.^16^ Thus, in these cases the problem persists in the form of risk of relapse after surgery. The optimal period for drug treatment, and the risk of long-term complications of the treatment are not established. We are not aware of any studies that have suggested an optimal period of calcium and phosphorus supplementation. Regular renal monitoring is advised to detect evidence of progressive renal calcification.

In conclusion, the oncogenic osteomalacia syndrome is caused by a protein secreted from oncogenic osteomalacia–producing mesenchymal tumors, and it manifests as osteomalacia with hypophosphatemia and hyperphosphaturia. Excision of the tumor causes improvement in general health and blood parameters. However, identification of the oncogenic osteomalacia-producing lesion is difficult, and this delays diagnosis and makes management lengthy. A high index of suspicion is needed to diagnose this entity.
